# 4-(4-Bromo­phen­yl)-1-(2,6-difluoro­benz­yl)-3-(3,4,5-trimeth­oxy­phen­yl)-1*H*-1,2,4-triazole-5(4*H*)-thione

**DOI:** 10.1107/S1600536811052330

**Published:** 2011-12-10

**Authors:** Hoong-Kun Fun, Chin Wei Ooi, B. Chandrakantha, Arun M. Isloor, Prakash Shetty

**Affiliations:** a X-ray Crystallography Unit, School of Physics, Universiti Sains Malaysia, 11800 USM, Penang, Malaysia; b Department of Chemistry, Manipal Institute of Technology, Manipal 576 104, India; c Medicinal Chemistry Division, Department of Chemistry, National Institute of Technology-Karnataka, Surathkal, Mangalore 575 025, India; d Department of Printing, Manipal Institute of Technology, Manipal 576 104, India

## Abstract

In the title compound, C_24_H_20_BrF_2_N_3_O_3_S, the triazole ring (r.m.s. deviation = 0.0107 Å) makes dihedral angles of 28.18 (14), 63.76 (14) and 77.01 (18)°, respectively, with the trimeth­oxy-, bromo-, and difluoro-substituted benzene rings. The C atoms of the *meta* meth­oxy groups are roughly coplanar with their ring [displacements = −0.289 (4) and 0.083 (7) Å], whereas the C atom of the *para* group is displaced [1.117 (3) Å]. In the crystal, inversion dimers linked by two pairs of C—H⋯O hydrogen bonds occur. The ring motif of the two hydrogen bonds to their symmetry-generated O-atom acceptors is *R*_2_^2^(8).

## Related literature

For a related structure and background to 1,2,4-triazole derivatives, see: Fun *et al.* (2011 ▶). For hydrogen-bond motifs, see: Bernstein *et al.* (1995 ▶).
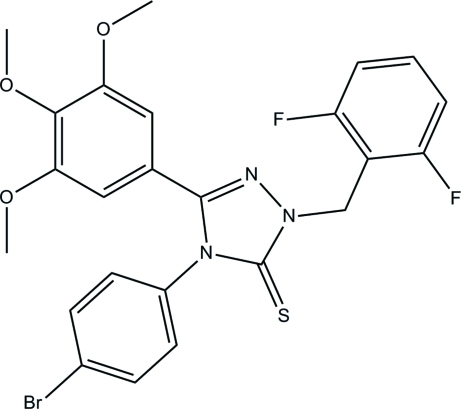


## Experimental

### 

#### Crystal data

C_24_H_20_BrF_2_N_3_O_3_S
*M* *_r_* = 548.40
Monoclinic, 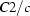

*a* = 17.6694 (18) Å
*b* = 15.5299 (16) Å
*c* = 18.0855 (19) Å
β = 102.955 (2)°
*V* = 4836.4 (9) Å^3^
*Z* = 8
Mo *K*α radiation
μ = 1.83 mm^−1^
*T* = 296 K
0.63 × 0.34 × 0.21 mm

#### Data collection

Bruker APEX DUO CCD diffractometer
Absorption correction: multi-scan (*SADABS*; Bruker, 2009 ▶) *T*_min_ = 0.391, *T*_max_ = 0.703
20567 measured reflections
7051 independent reflections
3435 reflections with *I* > 2σ(*I*)
*R*_int_ = 0.041

#### Refinement

*R*[*F* ^2^ > 2σ(*F* ^2^)] = 0.048
*wR*(*F* ^2^) = 0.158
*S* = 1.00
7051 reflections
310 parameters
H-atom parameters constrained
Δρ_max_ = 0.30 e Å^−3^
Δρ_min_ = −0.44 e Å^−3^

### 

Data collection: *APEX2* (Bruker, 2009 ▶); cell refinement: *SAINT* (Bruker, 2009 ▶); data reduction: *SAINT*; program(s) used to solve structure: *SHELXTL* (Sheldrick, 2008 ▶); program(s) used to refine structure: *SHELXTL*; molecular graphics: *SHELXTL*; software used to prepare material for publication: *SHELXTL* and *PLATON* (Spek, 2009 ▶).

## Supplementary Material

Crystal structure: contains datablock(s) global, I. DOI: 10.1107/S1600536811052330/hb6551sup1.cif

Structure factors: contains datablock(s) I. DOI: 10.1107/S1600536811052330/hb6551Isup2.hkl

Supplementary material file. DOI: 10.1107/S1600536811052330/hb6551Isup3.cml

Additional supplementary materials:  crystallographic information; 3D view; checkCIF report

## Figures and Tables

**Table 1:** Hydrogen-bond geometry (Å, °)

*D*—H⋯*A*	*D*—H	H⋯*A*	*D*⋯*A*	*D*—H⋯*A*
C13—H13*A*⋯O1^i^	0.93	2.54	3.284 (3)	137
C14—H14*A*⋯O2^i^	0.93	2.40	3.142 (3)	137

Symmetry code: (i) 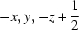.

**Fig. 1.**
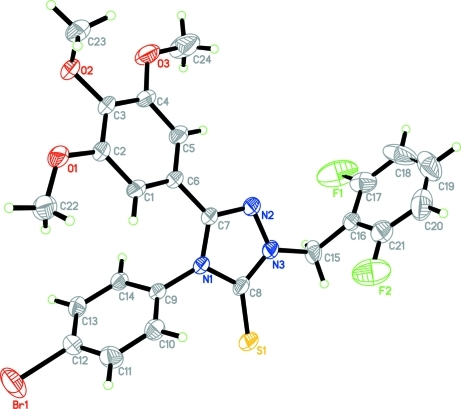
The molecular structure of the title compound, showing 30% probability displacement ellipsoids.

**Fig. 2.**
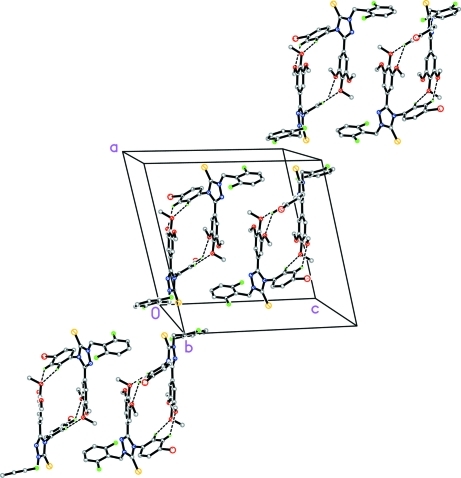
The crystal packing of the title compound, viewed down b axis. Dashed lines represent hydrogen bonds.

## supplementary crystallographic information

### Comment

As part of our ongoing studies of 1,2,4-triazole derivatives (Fun *et al.*, 2011), we now describe the structure of the title compound, (I).

In the title compound (Fig. 1), the triazole (N1–N3/C7/C8) ring is essentially planar with maximum deviation of 0.015 (3) Å at atom C8. The central triazole ring makes dihedral angles of 28.18 (14)°, 63.76 (14)° and 77.01 (18)° respectively with the methoxy (C1–C6), bromo (C9–C14), and difluoro (C16–C21) substituted phenyl rings. The bond lengths are comparable to a related structure (Fun *et al.*, 2011).

In the crystal (Fig. 2), the intermolecular C13—H13A···O1 and C14—H14A···O2 hydrogen bonds (Table 1) link the molecules to form *R*_2_^2^ (8) ring motifs (Bernstein *et al.*, 1995), leading to the formation of dimers.

### Experimental

To a solution of 4-(4-bromophenyl)-5-(3,4,5-trimethoxyphenyl)-4*H*-1,2,4- triazole-3-thiol (1 g, 0.0023 mol) in dry acetonitrile (20 ml) was added potassium carbonate (0.65 g, 0.0047 mol) followed by 2,6-difluorobenzyl bromide (0.52 g, 0.0025 mol) at room temperature. After the addition, the reaction mixture was stirred at room temperature for 6 hr. Progress of reaction was monitored by TLC. After the completion of the reaction, the reaction mixture was concentrated and purified by column chromatography using pet ether, ethyl acetate as an eluent to afford title compound as a colourless solid. Yield 1.1 g, 85%. *M*.p: 448–453 K.

### Refinement

All the H atoms were positioned geometrically and refined using a riding model with *U*_iso_(H) = 1.2 or 1.5*U*_eq_(C) (C—H = 0.93, 0.96 or 0.97 Å). A rotating group model was applied to the methyl group. In the final refinement, one outliner (-2 0 8) was omitted.

### Crystal data

**Table:**

C_24_H_20_BrF_2_N_3_O_3_S	*F*(000) = 2224
*M**_r_* = 548.40	*D*_x_ = 1.506 Mg m^−^^3^
Monoclinic, *C*2/*c*	Mo *K*α radiation, λ = 0.71073 Å
Hall symbol: -C 2yc	Cell parameters from 3384 reflections
*a* = 17.6694 (18) Å	θ = 2.3–22.8°
*b* = 15.5299 (16) Å	µ = 1.83 mm^−^^1^
*c* = 18.0855 (19) Å	*T* = 296 K
β = 102.955 (2)°	Block, colourless
*V* = 4836.4 (9) Å^3^	0.63 × 0.34 × 0.21 mm
*Z* = 8	

### Data collection

**Table:**

Bruker APEX DUO CCD diffractometer	7051 independent reflections
Radiation source: fine-focus sealed tube	3435 reflections with *I* > 2σ(*I*)
graphite	*R*_int_ = 0.041
φ and ω scans	θ_max_ = 30.1°, θ_min_ = 1.8°
Absorption correction: multi-scan (*SADABS*; Bruker, 2009)	*h* = −23→24
*T*_min_ = 0.391, *T*_max_ = 0.703	*k* = −21→20
20567 measured reflections	*l* = −25→25

### Refinement

**Table:**

Refinement on *F*^2^	Primary atom site location: structure-invariant direct methods
Least-squares matrix: full	Secondary atom site location: difference Fourier map
*R*[*F*^2^ > 2σ(*F*^2^)] = 0.048	Hydrogen site location: inferred from neighbouring sites
*wR*(*F*^2^) = 0.158	H-atom parameters constrained
*S* = 1.00	*w* = 1/[σ^2^(*F*_o_^2^) + (0.0774*P*)^2^] where *P* = (*F*_o_^2^ + 2*F*_c_^2^)/3
7051 reflections	(Δ/σ)_max_ < 0.001
310 parameters	Δρ_max_ = 0.30 e Å^−^^3^
0 restraints	Δρ_min_ = −0.44 e Å^−^^3^

### Special details

**Table:**

Geometry. All e.s.d.'s (except the e.s.d. in the dihedral angle between two l.s. planes) are estimated using the full covariance matrix. The cell e.s.d.'s are taken into account individually in the estimation of e.s.d.'s in distances, angles and torsion angles; correlations between e.s.d.'s in cell parameters are only used when they are defined by crystal symmetry. An approximate (isotropic) treatment of cell e.s.d.'s is used for estimating e.s.d.'s involving l.s. planes.
Refinement. Refinement of *F*^2^ against ALL reflections. The weighted *R*-factor *wR* and goodness of fit *S* are based on *F*^2^, conventional *R*-factors *R* are based on *F*, with *F* set to zero for negative *F*^2^. The threshold expression of *F*^2^ > σ(*F*^2^) is used only for calculating *R*-factors(gt) *etc*. and is not relevant to the choice of reflections for refinement. *R*-factors based on *F*^2^ are statistically about twice as large as those based on *F*, and *R*- factors based on ALL data will be even larger.

### Fractional atomic coordinates and isotropic or equivalent isotropic displacement parameters (Å^2^)

**Table:**

	*x*	*y*	*z*	*U*_iso_*/*U*_eq_	
Br1	0.20988 (3)	0.55993 (2)	0.21898 (3)	0.1130 (2)	
S1	0.42133 (3)	0.87749 (5)	0.42274 (5)	0.0673 (2)	
F1	0.32718 (17)	1.22282 (19)	0.4955 (2)	0.1545 (12)	
F2	0.42211 (15)	0.98693 (17)	0.64170 (17)	0.1319 (10)	
O1	−0.02973 (10)	0.76975 (11)	0.37056 (14)	0.0706 (6)	
O2	−0.11356 (9)	0.91409 (13)	0.34642 (12)	0.0649 (5)	
O3	−0.04768 (11)	1.06802 (12)	0.37062 (18)	0.0906 (8)	
N1	0.26407 (10)	0.88186 (13)	0.41197 (11)	0.0444 (5)	
N2	0.24694 (11)	1.00640 (14)	0.46509 (13)	0.0564 (6)	
N3	0.32475 (11)	0.99013 (14)	0.46984 (13)	0.0557 (6)	
C1	0.09112 (12)	0.85034 (16)	0.40263 (15)	0.0498 (6)	
H1A	0.1210	0.8005	0.4104	0.060*	
C2	0.01028 (13)	0.84511 (16)	0.38229 (15)	0.0520 (6)	
C3	−0.03423 (13)	0.91950 (17)	0.37176 (16)	0.0536 (6)	
C4	0.00131 (13)	0.99918 (17)	0.38142 (18)	0.0609 (7)	
C5	0.08213 (14)	1.00515 (17)	0.40030 (18)	0.0599 (7)	
H5A	0.1062	1.0588	0.4056	0.072*	
C6	0.12633 (13)	0.93048 (15)	0.41108 (15)	0.0492 (6)	
C7	0.21156 (13)	0.94011 (15)	0.43001 (15)	0.0487 (6)	
C8	0.33770 (12)	0.91620 (17)	0.43633 (14)	0.0493 (6)	
C9	0.24939 (12)	0.80534 (15)	0.36716 (13)	0.0428 (5)	
C10	0.27705 (15)	0.72710 (18)	0.39855 (16)	0.0595 (7)	
H10A	0.3036	0.7241	0.4491	0.071*	
C11	0.26513 (18)	0.65378 (18)	0.35485 (18)	0.0694 (8)	
H11A	0.2838	0.6010	0.3754	0.083*	
C12	0.22515 (15)	0.65947 (17)	0.28023 (16)	0.0577 (7)	
C13	0.19658 (14)	0.73658 (17)	0.24914 (15)	0.0544 (6)	
H13A	0.1687	0.7391	0.1991	0.065*	
C14	0.20950 (13)	0.81059 (15)	0.29267 (13)	0.0468 (6)	
H14A	0.1914	0.8634	0.2718	0.056*	
C15	0.38362 (16)	1.0561 (2)	0.49590 (19)	0.0726 (9)	
H15A	0.3819	1.0975	0.4554	0.087*	
H15B	0.4344	1.0292	0.5063	0.087*	
C16	0.37404 (13)	1.10289 (16)	0.56521 (15)	0.0505 (6)	
C17	0.34767 (18)	1.1854 (2)	0.5644 (2)	0.0794 (10)	
C18	0.3428 (3)	1.2317 (3)	0.6272 (5)	0.125 (2)	
H18A	0.3239	1.2878	0.6236	0.149*	
C19	0.3670 (3)	1.1915 (4)	0.6960 (4)	0.130 (2)	
H19A	0.3644	1.2215	0.7399	0.156*	
C20	0.3937 (2)	1.1119 (4)	0.7023 (2)	0.1091 (15)	
H20A	0.4101	1.0860	0.7495	0.131*	
C21	0.39669 (18)	1.0688 (2)	0.6371 (2)	0.0739 (9)	
C22	0.01256 (18)	0.69298 (18)	0.3665 (2)	0.0775 (9)	
H22A	−0.0230	0.6465	0.3499	0.116*	
H22B	0.0441	0.6799	0.4158	0.116*	
H22C	0.0453	0.7006	0.3312	0.116*	
C23	−0.15565 (16)	0.9151 (2)	0.4041 (2)	0.0742 (8)	
H23A	−0.2103	0.9147	0.3816	0.111*	
H23B	−0.1428	0.9661	0.4343	0.111*	
H23C	−0.1425	0.8652	0.4357	0.111*	
C24	−0.0154 (2)	1.1508 (2)	0.3820 (4)	0.1291 (19)	
H24A	−0.0563	1.1929	0.3738	0.194*	
H24B	0.0173	1.1606	0.3470	0.194*	
H24C	0.0148	1.1557	0.4330	0.194*	

### Atomic displacement parameters (Å^2^)

**Table:**

	*U*^11^	*U*^22^	*U*^33^	*U*^12^	*U*^13^	*U*^23^
Br1	0.1658 (5)	0.0668 (2)	0.0968 (3)	0.0079 (2)	0.0091 (3)	−0.0314 (2)
S1	0.0325 (3)	0.0908 (5)	0.0767 (5)	0.0032 (3)	0.0079 (3)	−0.0227 (4)
F1	0.127 (2)	0.136 (2)	0.212 (3)	0.0421 (17)	0.065 (2)	0.094 (2)
F2	0.130 (2)	0.1086 (17)	0.160 (2)	0.0429 (15)	0.0390 (18)	0.0481 (17)
O1	0.0405 (9)	0.0573 (11)	0.1115 (17)	−0.0094 (8)	0.0114 (10)	−0.0154 (11)
O2	0.0308 (8)	0.0846 (12)	0.0747 (13)	−0.0053 (8)	0.0021 (8)	−0.0160 (11)
O3	0.0420 (10)	0.0622 (13)	0.159 (2)	0.0074 (9)	0.0055 (13)	−0.0095 (13)
N1	0.0295 (8)	0.0537 (11)	0.0469 (11)	−0.0002 (8)	0.0022 (8)	−0.0066 (9)
N2	0.0328 (9)	0.0679 (13)	0.0687 (14)	−0.0089 (9)	0.0119 (10)	−0.0213 (11)
N3	0.0308 (9)	0.0750 (14)	0.0608 (13)	−0.0074 (9)	0.0094 (9)	−0.0233 (12)
C1	0.0336 (11)	0.0548 (14)	0.0599 (16)	0.0018 (10)	0.0084 (11)	−0.0019 (12)
C2	0.0350 (11)	0.0592 (15)	0.0600 (16)	−0.0068 (11)	0.0068 (11)	−0.0103 (12)
C3	0.0296 (11)	0.0649 (15)	0.0623 (16)	−0.0018 (10)	0.0015 (11)	−0.0110 (13)
C4	0.0348 (12)	0.0562 (15)	0.087 (2)	0.0059 (11)	0.0041 (13)	−0.0115 (14)
C5	0.0379 (12)	0.0535 (14)	0.085 (2)	−0.0026 (11)	0.0075 (13)	−0.0096 (14)
C6	0.0303 (10)	0.0581 (14)	0.0578 (15)	−0.0025 (10)	0.0068 (10)	−0.0109 (12)
C7	0.0326 (11)	0.0568 (14)	0.0549 (15)	−0.0021 (10)	0.0063 (10)	−0.0113 (12)
C8	0.0308 (11)	0.0706 (16)	0.0440 (13)	−0.0032 (10)	0.0034 (10)	−0.0065 (12)
C9	0.0314 (10)	0.0508 (13)	0.0445 (13)	0.0033 (9)	0.0050 (10)	−0.0043 (11)
C10	0.0605 (15)	0.0619 (16)	0.0481 (15)	0.0093 (13)	−0.0046 (12)	0.0023 (13)
C11	0.082 (2)	0.0491 (15)	0.071 (2)	0.0130 (14)	0.0040 (16)	0.0051 (14)
C12	0.0579 (15)	0.0527 (14)	0.0610 (18)	0.0018 (12)	0.0101 (13)	−0.0101 (13)
C13	0.0507 (14)	0.0624 (16)	0.0452 (14)	−0.0022 (12)	0.0007 (11)	−0.0021 (12)
C14	0.0412 (12)	0.0486 (13)	0.0470 (14)	0.0024 (10)	0.0020 (11)	0.0019 (11)
C15	0.0457 (14)	0.099 (2)	0.077 (2)	−0.0327 (14)	0.0223 (14)	−0.0379 (17)
C16	0.0325 (11)	0.0570 (15)	0.0620 (17)	−0.0094 (10)	0.0105 (11)	−0.0123 (13)
C17	0.0631 (18)	0.0624 (18)	0.120 (3)	−0.0035 (15)	0.0364 (19)	0.007 (2)
C18	0.091 (3)	0.065 (2)	0.245 (7)	−0.025 (2)	0.095 (4)	−0.060 (4)
C19	0.088 (3)	0.165 (6)	0.155 (5)	−0.062 (4)	0.062 (4)	−0.102 (5)
C20	0.076 (2)	0.184 (5)	0.067 (2)	−0.029 (3)	0.0154 (19)	−0.026 (3)
C21	0.0535 (16)	0.088 (2)	0.080 (2)	−0.0048 (15)	0.0135 (15)	−0.0103 (19)
C22	0.0619 (17)	0.0548 (16)	0.112 (3)	−0.0076 (14)	0.0124 (17)	−0.0017 (17)
C23	0.0445 (14)	0.085 (2)	0.095 (2)	−0.0043 (14)	0.0199 (16)	−0.0046 (19)
C24	0.067 (2)	0.056 (2)	0.251 (6)	0.0080 (16)	0.009 (3)	0.000 (3)

### Geometric parameters (Å, °)

**Table:**

Br1—C12	1.885 (3)	C10—H10A	0.9300
S1—C8	1.664 (2)	C11—C12	1.379 (4)
F1—C17	1.349 (5)	C11—H11A	0.9300
F2—C21	1.344 (4)	C12—C13	1.370 (4)
O1—C2	1.359 (3)	C13—C14	1.383 (3)
O1—C22	1.418 (3)	C13—H13A	0.9300
O2—C3	1.376 (3)	C14—H14A	0.9300
O2—C23	1.411 (4)	C15—C16	1.491 (4)
O3—C4	1.362 (3)	C15—H15A	0.9700
O3—C24	1.403 (4)	C15—H15B	0.9700
N1—C8	1.384 (3)	C16—C17	1.363 (4)
N1—C7	1.386 (3)	C16—C21	1.378 (4)
N1—C9	1.429 (3)	C17—C18	1.363 (7)
N2—C7	1.294 (3)	C18—C19	1.372 (8)
N2—N3	1.381 (3)	C18—H18A	0.9300
N3—C8	1.342 (3)	C19—C20	1.319 (7)
N3—C15	1.461 (3)	C19—H19A	0.9300
C1—C6	1.384 (3)	C20—C21	1.367 (6)
C1—C2	1.395 (3)	C20—H20A	0.9300
C1—H1A	0.9300	C22—H22A	0.9600
C2—C3	1.386 (3)	C22—H22B	0.9600
C3—C4	1.381 (4)	C22—H22C	0.9600
C4—C5	1.395 (3)	C23—H23A	0.9600
C5—C6	1.387 (3)	C23—H23B	0.9600
C5—H5A	0.9300	C23—H23C	0.9600
C6—C7	1.475 (3)	C24—H24A	0.9600
C9—C14	1.376 (3)	C24—H24B	0.9600
C9—C10	1.383 (3)	C24—H24C	0.9600
C10—C11	1.375 (4)		
			
C2—O1—C22	118.1 (2)	C12—C13—H13A	120.2
C3—O2—C23	114.8 (2)	C14—C13—H13A	120.2
C4—O3—C24	118.3 (2)	C9—C14—C13	119.4 (2)
C8—N1—C7	107.74 (19)	C9—C14—H14A	120.3
C8—N1—C9	122.63 (19)	C13—C14—H14A	120.3
C7—N1—C9	129.08 (17)	N3—C15—C16	114.0 (2)
C7—N2—N3	104.45 (19)	N3—C15—H15A	108.8
C8—N3—N2	113.36 (18)	C16—C15—H15A	108.8
C8—N3—C15	124.4 (2)	N3—C15—H15B	108.8
N2—N3—C15	121.2 (2)	C16—C15—H15B	108.8
C6—C1—C2	119.3 (2)	H15A—C15—H15B	107.7
C6—C1—H1A	120.3	C17—C16—C21	113.4 (3)
C2—C1—H1A	120.3	C17—C16—C15	123.5 (3)
O1—C2—C3	115.9 (2)	C21—C16—C15	122.8 (3)
O1—C2—C1	123.9 (2)	F1—C17—C18	119.2 (4)
C3—C2—C1	120.2 (2)	F1—C17—C16	115.8 (4)
O2—C3—C4	119.8 (2)	C18—C17—C16	124.9 (4)
O2—C3—C2	119.9 (2)	C17—C18—C19	116.8 (4)
C4—C3—C2	120.1 (2)	C17—C18—H18A	121.6
O3—C4—C3	115.4 (2)	C19—C18—H18A	121.6
O3—C4—C5	124.5 (2)	C20—C19—C18	122.5 (5)
C3—C4—C5	120.1 (2)	C20—C19—H19A	118.7
C6—C5—C4	119.5 (2)	C18—C19—H19A	118.7
C6—C5—H5A	120.3	C19—C20—C21	117.8 (5)
C4—C5—H5A	120.3	C19—C20—H20A	121.1
C1—C6—C5	120.7 (2)	C21—C20—H20A	121.1
C1—C6—C7	121.8 (2)	F2—C21—C20	119.1 (4)
C5—C6—C7	117.5 (2)	F2—C21—C16	116.4 (3)
N2—C7—N1	111.06 (19)	C20—C21—C16	124.5 (4)
N2—C7—C6	123.4 (2)	O1—C22—H22A	109.5
N1—C7—C6	125.6 (2)	O1—C22—H22B	109.5
N3—C8—N1	103.31 (19)	H22A—C22—H22B	109.5
N3—C8—S1	128.53 (17)	O1—C22—H22C	109.5
N1—C8—S1	128.1 (2)	H22A—C22—H22C	109.5
C14—C9—C10	120.7 (2)	H22B—C22—H22C	109.5
C14—C9—N1	119.6 (2)	O2—C23—H23A	109.5
C10—C9—N1	119.7 (2)	O2—C23—H23B	109.5
C11—C10—C9	119.8 (2)	H23A—C23—H23B	109.5
C11—C10—H10A	120.1	O2—C23—H23C	109.5
C9—C10—H10A	120.1	H23A—C23—H23C	109.5
C10—C11—C12	119.2 (2)	H23B—C23—H23C	109.5
C10—C11—H11A	120.4	O3—C24—H24A	109.5
C12—C11—H11A	120.4	O3—C24—H24B	109.5
C13—C12—C11	121.2 (2)	H24A—C24—H24B	109.5
C13—C12—Br1	119.0 (2)	O3—C24—H24C	109.5
C11—C12—Br1	119.8 (2)	H24A—C24—H24C	109.5
C12—C13—C14	119.6 (2)	H24B—C24—H24C	109.5
			
C7—N2—N3—C8	1.8 (3)	C15—N3—C8—S1	5.6 (4)
C7—N2—N3—C15	170.8 (3)	C7—N1—C8—N3	2.5 (3)
C22—O1—C2—C3	168.4 (3)	C9—N1—C8—N3	174.7 (2)
C22—O1—C2—C1	−10.7 (4)	C7—N1—C8—S1	−174.3 (2)
C6—C1—C2—O1	178.1 (3)	C9—N1—C8—S1	−2.1 (4)
C6—C1—C2—C3	−0.9 (4)	C8—N1—C9—C14	−110.9 (3)
C23—O2—C3—C4	−89.1 (3)	C7—N1—C9—C14	59.5 (3)
C23—O2—C3—C2	95.5 (3)	C8—N1—C9—C10	67.5 (3)
O1—C2—C3—O2	−3.8 (4)	C7—N1—C9—C10	−122.1 (3)
C1—C2—C3—O2	175.3 (2)	C14—C9—C10—C11	0.5 (4)
O1—C2—C3—C4	−179.2 (3)	N1—C9—C10—C11	−178.0 (2)
C1—C2—C3—C4	0.0 (4)	C9—C10—C11—C12	−0.4 (4)
C24—O3—C4—C3	177.9 (4)	C10—C11—C12—C13	−0.6 (5)
C24—O3—C4—C5	−2.6 (6)	C10—C11—C12—Br1	178.9 (2)
O2—C3—C4—O3	5.4 (4)	C11—C12—C13—C14	1.5 (4)
C2—C3—C4—O3	−179.2 (3)	Br1—C12—C13—C14	−177.93 (19)
O2—C3—C4—C5	−174.1 (3)	C10—C9—C14—C13	0.5 (4)
C2—C3—C4—C5	1.3 (5)	N1—C9—C14—C13	178.9 (2)
O3—C4—C5—C6	179.0 (3)	C12—C13—C14—C9	−1.5 (4)
C3—C4—C5—C6	−1.6 (5)	C8—N3—C15—C16	−146.7 (3)
C2—C1—C6—C5	0.7 (4)	N2—N3—C15—C16	45.6 (4)
C2—C1—C6—C7	−177.9 (2)	N3—C15—C16—C17	−106.4 (3)
C4—C5—C6—C1	0.6 (5)	N3—C15—C16—C21	78.7 (3)
C4—C5—C6—C7	179.2 (3)	C21—C16—C17—F1	177.9 (3)
N3—N2—C7—N1	−0.1 (3)	C15—C16—C17—F1	2.5 (4)
N3—N2—C7—C6	−178.9 (2)	C21—C16—C17—C18	−0.4 (4)
C8—N1—C7—N2	−1.6 (3)	C15—C16—C17—C18	−175.8 (3)
C9—N1—C7—N2	−173.1 (2)	F1—C17—C18—C19	−177.7 (4)
C8—N1—C7—C6	177.2 (3)	C16—C17—C18—C19	0.5 (6)
C9—N1—C7—C6	5.7 (4)	C17—C18—C19—C20	−0.1 (7)
C1—C6—C7—N2	−153.5 (3)	C18—C19—C20—C21	−0.3 (7)
C5—C6—C7—N2	27.9 (4)	C19—C20—C21—F2	−178.6 (3)
C1—C6—C7—N1	27.9 (4)	C19—C20—C21—C16	0.4 (6)
C5—C6—C7—N1	−150.7 (3)	C17—C16—C21—F2	178.9 (3)
N2—N3—C8—N1	−2.7 (3)	C15—C16—C21—F2	−5.7 (4)
C15—N3—C8—N1	−171.3 (3)	C17—C16—C21—C20	−0.1 (4)
N2—N3—C8—S1	174.1 (2)	C15—C16—C21—C20	175.3 (3)

### Hydrogen-bond geometry (Å, °)

**Table:**

*D*—H···*A*	*D*—H	H···*A*	*D*···*A*	*D*—H···*A*
C13—H13A···O1^i^	0.93	2.54	3.284 (3)	137
C14—H14A···O2^i^	0.93	2.40	3.142 (3)	137

Symmetry codes: (i) −*x*, *y*, −*z*+1/2.
